# Utilization of *Tenebrio molitor* Larvae Reared with Different Substrates as Feed Ingredients in Growing Pigs

**DOI:** 10.3390/vetsci10060393

**Published:** 2023-06-09

**Authors:** Christos Zacharis, Eleftherios Bonos, Ilias Giannenas, Ioannis Skoufos, Athina Tzora, Chrysoula (Chrysa) Voidarou, Anastasios Tsinas, Konstantina Fotou, Georgios Papadopoulos, Chrysanthi Mitsagga, Christos Athanassiou, Efthimia Antonopoulou, Katerina Grigoriadou

**Affiliations:** 1 Department of Agriculture, University of Ioannina, Kostakioi Artas, 47100 Arta, Greece; 2 School of Veterinary Medicine, Aristotle University of Thessaloniki, 54124 Thessaloniki, Greece; 3 Department of Food Science and Nutrition, School of Agricultural Sciences, University of Thessaly, 43100 Karditsa, Greece; 4 Department of Agriculture, Crop Production and Rural Environment, University of Thessaly, 38446 Nea Ionia, Greece; 5 Department of Biology, Aristotle University of Thessaloniki, 54124 Thessaloniki, Greece; 6 Institute of Plant Breeding and Genetic Resources, Hellenic Agricultural Organization ELGO-DIMITRA, 57001 Thessaloniki, Greece

**Keywords:** insects as feed, aromatic plants, swine, health, gut microbiota, meat quality

## Abstract

**Simple Summary:**

The procurement of adequate feed resources is one of the most important challenges for the animal industry worldwide. While the need for feeds rich in protein is constantly increasing, their production cannot readily keep up. Consequently, it is necessary to identify and develop new feeding strategies and feed ingredients, such as insect meals, to overcome this challenge in a sustainable way. In the present study, *Tenebrio molitor* larvae that were fed on either a standard substrate or enriched with aromatic plant material were used as feed ingredients for growing pigs. A total of 36 weaned pigs (34 days old) were allocated to three groups of 12 pigs each and fed for 42 days either a conventional feed (group A) or two feeds in which the larvae meals were included at 10% (groups B and C, respectively). The three groups had similar growth and feed consumption rates. Fecal samples were analyzed, and the results showed that some important bacterial families were found in different populations. Blood analysis showed that the insect supplementation modified total cholesterol but not the rest of the parameters. Meat cuts had different enumerations of microbial populations, higher amounts of total phenols, and different fatty acid profiles, whereas they were similar in their proximate analysis and color.

**Abstract:**

The procurement of adequate feed resources is one of the most important challenges for the animal industry worldwide. While the need for feeds rich in protein is constantly increasing, their production cannot readily keep up. Consequently, to overcome this challenge in a sustainable way, it is necessary to identify and develop new feeding strategies and feed ingredients, such as insect meals. In the present study, *Tenebrio molitor* larvae that were reared on two different substrates (standard and enriched with medicinal aromatic plant material) were used as feed ingredients for growing pigs. A total of 36 weaned pigs (34 days old) were randomly allocated to three treatment groups and fed either the control diet (A) or diets supplemented at 10% with one of the two insect meals (B and C). At the end of the trial (42 days), blood, feces, and meat samples were collected for analysis. The insect meal supplementation did not affect (*p* > 0.05) overall performance but significantly modified (*p* < 0.001) the fecal microflora balance and the blood cholesterol (*p* < 0.001), while the rest of the blood parameters tested were not affected. Moreover, this dietary supplementation significantly affected some microbial populations (*p* < 0.001), improved the total phenolic content (*p* < 0.05), and the fatty acid profile (*p* < 0.001) of the meat cuts, but did not affect (*p* > 0.05) meat color or proximate composition. Further research is needed to evaluate the different types and levels of inclusion of insect meals in pig nutrition.

## 1. Introduction

During the last few decades, there has been a large increase in the world’s human population, with a simultaneous improvement in its standard of living. To feed these people, increasing amounts of food are needed, both from plant and from animal origin [1,2]. Consequently, the future increase in global consumption is currently one of the most important challenges for the food industry and the management of available natural resources [3]. At the same time, these increased dietary needs should be realized through sustainable methods of production and development. For example, in the European Union, it is known that the production of domestic animal feeds is not sufficient, and as a consequence, large quantities of them are imported every year from America or Asia [4,5]. This shortage is particularly evident in protein feeds (soybean seeds, soybean meal, fish meals, etc.), which often have significant fluctuations in cost or availability. New land available for soybean cultivation is not easy to find, while marine overexploitation has dramatically minimized the abundance of small pelagic fish, which are used for fishmeal production. In fact, the cost and availability of soybean meal can change radically, as it is an agricultural commodity on the global market [6]. Additionally, the problem of feed sufficiency is further complicated by the strict restrictions on the use of by-products derived from the processing of animals’ tissues in the feed of farmed animals due to bovine spongiform encephalopathy [7,8,9]. Thus, identifying and utilizing new feeds with high quantities and quality of protein is an urgent need.

Insects, which are part of the natural diet of many species of fish, birds, and mammals, represent a new sustainable resource rich in protein [10], that can be used in human and livestock production [9], and can enhance food and feed security [11]. According to the directives of the EU Parliament (2008/98), insects are thought to be one of the most realistic solutions to reduce, reuse, recycle, and transform waste into high-quality raw materials [12,13]. Recent developments show that insects will have a significant role in the future as a protein source not only for their nutritional value but also for the comparatively lower environmental footprint of their production compared to many other farm animal species: lower production of greenhouse gases; lower consumption of water; and less cropland [14]. Furthermore, insects or products derived from them (protein meal and fats) can potentially be rich sources of bioactive compounds, such as antimicrobial peptides, fatty acids, and polysaccharides [15]. Recently, in April 2021, EU Member States voted positively on the authorization of insect processed animal proteins (PAPs) from eight insect species, i.e., *Hermetia illucens* (L.), *Musca domestica* (L.), *Tenebrio molitor* (L.), *Alphitobius diaperinus* (Panzer), *Acheta domesticus* (L.), *Gryllodes sigillatus* (Walker), *Gryllus assimilis* (F.), and *Bombyx mori* (L.) in poultry and pig feeds, and their use was allowed starting in 2021 [16].

According to the available published literature, insect species tissues can have widely different chemical compositions and different nutritional properties when used as food or feed [17,18,19]. Moreover, a large variability in composition can be observed even among the same insect species [18,20]. Furthermore, some studies have shown that the nutritional composition of insects can be affected by their diet [21], and maybe it is possible to improve their nutritional composition via diet [22]. The aim of the present study was to rear *T. molitor* larvae in two different substrates: one in a conventional substrate and one in a substrate enriched with residues of the distillation of medicinal aromatic plants that contain important bioactive compounds. Then, the grown *T. molitor* larvae were compared as feed ingredients (insect meals) in the diet of growing pigs. The impact of this dietary insect meal supplementation on the performance, gut microbiota, health status, and quality characteristics of the pigs’ meat was evaluated.

## 2. Materials and Methods

### 2.1. Experimental Design, Animals, and Diets

The experimental protocol for this trial was reviewed and approved by the Ethics and Research Ethics Committee of the University of Ioannina of Greece (protocol number 56652, 26 November 2021).

Thirty-six crossbreed weaned pigs (¼ Large White, ¼ Landrace, and ½ Duroc) 34 days old were selected from a commercial pig farm in the region of Epirus, Greece. Each pig was individually marked with ear tags. The average initial mean body weight of the pigs was 8.44 ± 0.83 kg. They were randomly allocated into 3 different groups (group A; group B; and group C), and each group was housed in separate pens with a slatted plastic floor. The environmental conditions (ambient temperature and humidity) were continuously monitored. Access to feed and water was ad libitum throughout the trial.

Two insect meals of *T. molitor* were used, which were reared on two different substrates. The first meal (“Conventional”) was created from insects reared in a conventional substrate, while the second meal (“Enriched”) was created from insects reared in a substrate partially enriched (20%) with plant material from residues of distillation of medicinal aromatic plants: Greek oregano (*Origanum vulgare* subsp. *hirtum*), thymus (*Thymus vulgaris*), sage (*Salvia officinalis*), rosemary (*Rosmarinus officinalis*) and their essential oils, linseed (*Linum usitatissimum*), sea fennel (*Crithmum maritimum*), and olive residues after the process. Insects were reared for a period of four months in total, starting from newly hatched larvae until the stage of late-instar larvae, i.e., prior to pupation, as suggested by Rumbos et al. [23], which was the instar that was used in the feeding trials. The insects were kept frozen (−20 °C) until being used for the preparation of the pig diets.

The control group (group A) was fed a commercial maize-barley-based diet, which was formulated according to the recommendations of the National Research Council [24] and the database of Premier Nutrition [25]. In the diet of group B, the “Conventional” meal was incorporated at 10%, whereas in the diet of group C, the “Enriched” meal was incorporated at 10%. The three diets were formulated to be isocaloric and isonitrogenous. The total phenolic content of the diets was analyzed with the Folin–Ciocalteu method as described by Vasilopoulos et al. [26]. Table 1 presents the ingredients and chemical composition of the three diets.

The whole experimental trial lasted 42 days. During the experimental period, all growing pigs were individually weighed on the 1st, 21st, and 42nd days using a Mini-L 3510 scale for animals (Zigisis, Chalkidiki, Greece). Feed intake and mortality data were collected daily. In addition, weight gain per pig, average feed intake per group, and average feed conversion ratio per group (FCR, kg feed intake/kg live weight gain) were calculated for the periods 1–21, 21–42, and 1–42 days of the trial. During the last day of the dietary trial, six pigs from each group were randomly selected and sacrificed in a local commercial slaughterhouse to collect tissues for further analysis.

### 2.2. Analysis of Fecal Microbiota through a Culture-Dependent Method

#### Isolation, Enumeration, and Identification of Bacteria in Fecal Samples

Fresh fecal (stool) samples were gathered on the last day (42nd) of the trial from each pig to analyze [27] and determine their bacterial profile. Initially, 1 g of a fresh fecal (stool) sample was homogenized with 9 mL of sterile peptone water solution at 0.1%. The Miles and Misra Plate Method (surface drop) was applied for the bacterial enumeration. The samples were serially diluted via 12-fold dilutions (from 10^−1^ to 10^−12^) using standard 96-well plates. Then, 10 µL of each dilution was inoculated on media and incubated properly. Specifically, total aerobic and anaerobic bacterial counts were determined using plate count agar medium (Oxoid, Basingstoke, UK), while plates were incubated at 30 °C aerobically for 48 h and at 37 °C anaerobically for 48–72 h, respectively. MacConkey and Kanamycin aesculin azide (KAA) agar (Merck, Darmstadt, Germany) were, respectively, used for the isolation of Enterobacteriaceae and Enterococcaceae. All plates were incubated aerobically at 37 °C for 24–48 h. De Man, Rogosa, and Sharpe (MRS) agar (Oxoid, Basingstoke, UK) and M17 agar (Lab M Limited, Lancashire, UK) were used for the isolation and enumeration of Lactobacillaceae, while media were incubated at 37 °C for 48 h in anaerobic conditions. For bacterial counts, typical colonies from an appropriate dilution were counted, and counts were expressed as colony-forming units (CFU) × log per 1 g wet weight sample. Typical colonies grown on different media were then described and subcultured. All bacterial populations were identified at family level by the automated Vitek 2 compact system (bioMérieux, Marcy l’Etoile, France), which provides reliable and accurate results for a wide range of Gram-positive and Gram-negative bacteria [28]. For the identification of Enterobacteriaceae, Enterococcaceae, and Lactobacillaceae, the Vitek 2 Gram-Negative identification card (ID-GN) (bioMérieux, Marcy l’Etoile, France), the Vitek 2 Gram-Positive identification card (ID-GP) (bioMérieux, Marcy l’Etoile, France), the CBC and ANC identification cards (bioMérieux, Marcy l’Etoile, France), and the Vitek 2 ANC ID card (bioMérieux, Marcy l’Etoile, France) were used, respectively.

### 2.3. Blood Analysis

On the last day of the dietary trial, the feeds were removed from the feeders 4 h before blood sampling. For the determination of hematological and biochemical parameters, blood samples were taken from six growing pigs per treatment prior to slaughter. For blood collection, 4 mL of blood was collected from the jugular vein of the pigs and placed in vacutainer tubes with ethylenediaminetetraacetic acid (EDTA). Hematological parameters (WBC, White Blood Cells; Lym, Lymphocytes; Mon, Monocytes; Gra, Granulocytes; RBC, Red Blood Cells; Hct, Hematocrit; Hb, Hemoglobin; and THR, Thrombomodulin) were determined using an automated analyzer MS4 (Melet Schloesing Lab, Osny, France) and biochemical parameters (ALB, Albumine; ALT, Alanine aminotransferase; AST, Aspartate aminotransferase; CHOL, Cholesterol; CK, Creatine kinase; GLU, Glucose; TBIL, Total Bilirubin; and TRIG, Triglycerides) in serum using the IDEXX VETTEST 8008 (IDEXX LAB, Westbrook, ME, USA).

### 2.4. Meat Cut Sample Collection

On the last day of the trial, the pigs were transported to a nearby commercial slaughterhouse and processed according to the national regulations (PD, 2013). Samples of meat from the ham (*biceps femoris* and *semimembranosus muscles*), shoulder (*trapezius* and *triceps branchi muscles*), belly (*external abdominal* and *oblique muscles*), and boneless steak (*longissimus thoracis*) were collected for further processing.

### 2.5. Meat Microbial Analysis

Microbial populations were identified and enumerated in meat samples from shoulder, belly, and boneless steak samples. From each sample, 10 g of meat were collected and homogenized in a Bagmixer 400 (Interscience, Saint-Nom-la-Bretèche, France) with 90 mL of sterile Maximum Recovery Diluent (MRD) (Oxoid, Basingstoke, UK). Each sample was 10-fold diluted using glass tubes with 9 mL of sterile MRD. From the appropriate dilution, either 1 mL or 0.1 mL were inoculated in Petri dishes for the enumeration of the bacterial counts. The tested microorganisms were: *Escherichia coli*, which was cultivated on Tryptone Bile X-Glucuronide (TBX) agar (Oxoid, Basingstoke, UK) and incubated aerobically at 37 °C for 24 h; Sulfite-Reducing Clostridia, which were cultivated on Perfringens Agar Base (Oxoid, Basingstoke, UK) and incubated at 37 °C for 48 h under anaerobic conditions using anaerobic jars with the addition of Anaerocult A (Oxoid, Basingstoke, UK); *Staphylococcus aureus* and *Staphylococcus* sp. That were spread on Baird Parker agar (Oxoid, Basingstoke, UK), which was supplemented with egg yolk tellurite (50 mL/1 L substrate) and incubated under aerobic conditions at 37 °C for 48 h; Total Mesophilic Counts that were measured in Plate Count Agar (PCA) (Oxoid, Basingstoke, UK) at 30 °C for 48 h under aerobic conditions; and *Campylobacter jejuni* that was spread on Campy Blood Free Selective Medium (CCDA) (Acumedia–Lab M, Lansing, MI, USA) with Campylobacter selective supplement under microaerophilic conditions in an incubator with 10% CO_2_ at 37 °C for 72 h. All samples were examined for the presence of *Salmonella* spp. And *Listeria monocytogenes* per 25 g of meat using, respectively, the ISO 6579:2002 and ΙSO 4833:2001 methods [29,30]. The Petri dishes were incubated in Binder BD 115 thermostable incubators [31].

### 2.6. Meat Chemical Analysis

For the determination of meat chemical analysis, all meat samples that were collected during scarification were stored at −20 °C. Samples of 200 g of meat were ground using an industrial large meat grinder (Bosch, Gerlingen, Germany). Moisture, crude protein, fat, collagen, and ash composition were determined by near infrared spectroscopy with the use of a FoodScanTM Lab (FOSS, Hillerod, Denmark) in transmittance mode, according to AOAC 2007.04 for meat and meat products [32,33].

### 2.7. Meat Total Polyphenols Analysis

For the measurement of the total polyphenols of the meat samples (shoulder, belly, and boneless steak), a modified Folin–Ciocalteu method was used [34]. According to this method, 0.2 g/L of gallic acid (Merck, Darmstadt, Germany) was diluted in 100 mL of distilled water. The stock solution was used to prepare the standard solutions of 0.005, 0.01, 0.05, 0.1, 0.25, 0.5, and 1 g/L of gallic acid. From each standard solution, 0.2 mL was transferred into a 50 ml falcon tube and mixed with 10.8 mL of distilled water, 8 mL of Na_2_CO_3_ (75 g Na_2_CO_3_ in 1 L distilled water) (Penta Chemicals, Prague, Czech Republic), and 1 mL of the Folin–Ciocalteu reagent (PanReac AppliChem, Darmstadt, Germany). A control sample was prepared in which 0.2 mL of distilled water was added instead of a standard solution to calibrate the UV-Vis spectrophotometer (DR 5000, Hach Lange, Ames, IA, USA). All tubes were homogenized in a vortex, and they were placed in a dark cabinet for 1 h at room temperature. After the incubation, the control was used to calibrate the UV-Vis spectrophotometer (DR 5000, Hach Lange) at 750 nm, and then all the standard solutions were measured. A standard curve of concentration of gallic acid and absorbance was constructed using Microsoft Excel software, and the R^2^ was 0.9989. The above procedure was followed to measure the total polyphenols in the meat.

Then, 5 g of shoulder, belly, or boneless steak meat were homogenized in a blender with 10 mL of distilled water and filtered with filter paper. A quantity of 0.2 mL of the filtrate was transferred into 50 mL falcon tubes and mixed with 10.8 mL of distilled water, 8 mL of Na_2_CO_3_ (75 g/L solution), and 1 mL of the Folin–Ciocalteu reagent. A blank sample was prepared in which 0.2 mL was added instead of the sample in order to calibrate the UV-Vis spectrophotometer. All tubes were mixed in a vortex and placed in a dark cabinet at room temperature for 1 h. After the incubation, the blank sample was used to calibrate the spectrophotometer at 750 nm, and then all the samples were measured.

### 2.8. Meat Oxidative Stability Analysis

For the measurement of lipid oxidation in the meat, a modified method by Dias et al. [35] was used. Shoulder, belly, and boneless steak meat cuts were used to measure lipid oxidation using the 2-thiobarbituric acid method (TBARS). From each sample, 5 g of meat was homogenized with 25 mL of trichloroacetic acid in a blender, transferred into a glass bottle, and left for 20 min. Then, the samples were filtered with filter paper, and 5 mL of the filtrate was transferred into glass tubes with 5 mL of 2-thiobarbituric acid. A blank sample was prepared, replacing the sample with 5 mL of trichloroacetic acid. All tubes were mixed in a vortex and placed in a water bath at 60 °C for 15 min. The samples were measured in a UV-Vis spectrophotometer after calibration with the blank sample at 532 nm.

### 2.9. Meat Color and pH Analysis

The color measurement of the shoulder, belly, and boneless steak meat samples was determined according to the “Hunter scale” (L, a, and b values) by using a CAM-System 500 (Lovibond, Amesbury, UK) according to the standard procedure [31].

The pH measurement of shoulder, belly, and boneless steak meat was performed using a portable Hanna instrument (Woonsocket, RI, USA) pH meter for solid samples [36].

### 2.10. Meat Fatty Acid Analysis

For shoulder and belly meat fatty acid analysis, samples were processed as described by O’Fallon et al. [37]. Separation and quantification of the methyl esters were performed using the method described by Skoufos et al. [38], with the use of TraceGC (Model K07332, Thermofinigan, Thermoquest, Milan, Italy) equipped with a flame ionization detector.

### 2.11. Statistical Analysis

The basic study design was a RCB (random complete block design), and each ear-tagged pig was considered an experimental unit. Log-transformation (log10) of microbiology data was performed prior to analysis. Data homogeneity was tested using Levene’s test. Experimental data were analyzed by one-way analysis of variance (one-way ANOVA) or the Krushar–Wallis test, depending on the data format, using the SPSS v20 statistical package (IBM, Armonk, NY, USA) [39]. The Tukey’s test was used for post-hoc comparisons between the three treatment groups. The significance level for all tests was set at 5% (*p* ≤ 0.05). Values of *p* between 0.05 and 0.10 (0.05 < *p* ≤ 0.10) were considered to have tendencies to differ.

## 3. Results

### 3.1. Total Phenolic Count

The total phenolic content value of the feed of control group A was 30.71 mg of gallic acid equivalents/L of extract. The feed of group B, which contained the “Conventional” *T. molitor* meal, showed a total phenolic content value of 47.67 mg of gallic acid equivalents/L of extract. The feed of group C, which contained the “Enriched” *T. molitor* meal, showed a total phenolic content value of 28.05 mg/L of extract.

### 3.2. Performance Parameters

The results of the examined diets on the performance parameters of the growing pigs are presented in Table 2. It was noted that group B had significantly increased (*p* ≤ 0.05) body weight on day 21 and significantly increased body weight gain (*p* ≤ 0.01) for the period of 1 to 21 days compared to the control group A; however, the groups did not differ (*p* > 0.10) at the end of the trial on day 42. The feed intake and feed conversion ratio were within the expected ranges for the commercial pig farm that housed the experimental trial.

Concerning the carcass parameters, group C had a tendency (0.05 < *p* ≤ 0.10) for increased carcass weight compared to control group A. The carcass dressing percentage did not differ (*p* > 0.10) between the three groups.

### 3.3. Fecal Microflora

The fecal microbiota was affected by insect meal supplementation (Table 3). On day 42, group C had significantly lower (*p* < 0.001) total aerobes compared to the other two groups. Moreover, group B tended to have lower Lactobacillaceae (0.05 < *p* ≤ 0.10) compared to control group A. The other examined bacterial populations (Enterobacteriaceae, Enterococcaceae, and total anaerobes) did not differ (*p* > 0.10) between the three groups.

### 3.4. Blood Parameters

Table 4 shows the effects of dietary supplementation on the examined hematological and biochemical parameters. In the hematological parameters, no significant differences (*p* > 0.10) were observed between the three groups. Concerning the biochemical parameters, the cholesterol level of group C was significantly higher (*p* ≤ 0.001) compared to the other two groups, whereas the other examined biochemical parameters did not differ (*p* > 0.10).

### 3.5. Microbiological, Chemical, and Oxidative Stability Analysis of the Meat

The microbial analysis of the three different meat cuts is given in Table 5. In shoulder meat, groups A and C had significantly higher (*p* ≤ 0.05) Escherichia coli and Clostridium spp. counts compared to group B. Furthermore, Staphylococcus spp. were higher (*p* ≤ 0.05) in group A compared to group B. In belly meat, Clostridium spp. tended to be higher (0.05 < *p* ≤ 0.10) in group C compared to group B. Moreover, the microbiota of boneless steak meat did not differ (*p* > 0.10) between the three dietary groups. Finally, in the meat of all three groups, there was an absence of Salmonella sp. and Listeria monocytogenes (per 25 g of samples).

Regarding the chemical composition analysis of the meat cuts (Table 6), the only significant finding was that the collagen of the ham meat cut was significantly decreased (*p* ≤ 0.05) in group B compared to group A. All the other examined meat chemical composition parameters (fat, protein, moisture, and ash) did not differ (*p* > 0.010) between the three groups.

Table 7 presents the results of the dietary supplementation on the meat’s total phenolic content, oxidative stability, pH, and color. The shoulder meat of group B had significantly higher (*p* ≤ 0.05) total phenols compared to group A. Additionally, the boneless steak meat of groups B and C had significantly higher (*p* ≤ 0.05) total phenols compared to group A. Furthermore, the belly meat of group C tended to have higher (0.05 < *p* ≤ 0.1) total phenols compared to group A. The oxidative stability analysis of the shoulder meat showed that group B had significantly lower (*p* ≤ 0.05) shoulder meat TBARS compared to group A. However, in belly and boneless steak meat, no significant differences (*p* > 0.10) were observed between the three groups. In addition, the other examined meat parameters (pH and color L*, A*, and B*) did not differ significantly (*p* > 0.10) between the three treatments.

The effects of dietary supplementation on the shoulder meat fatty acid profile are presented in Table 8. C14:0 (Myristic) fatty acid was lower (*p* ≤ 0.01) in groups B and C compared to group A. C15:0 (Pentadecanoic) fatty acid tended to be lower (0.05 < *p* ≤ 0.10) in group B compared to group A. C16:1 (Palmitoleic) fatty acid was higher (*p* ≤ 0.01) in groups B and C compared to control group A. C17:1 (cis-10-Heptadecanoic) fatty acid tended to be higher (0.05 < *p* ≤ 0.10) in group B compared to group C. C18:1n9t (Elaidic) fatty acid tended to be higher (0.05 < *p* ≤ 0.10) in group C compared to group A. C18:1n9c (Oleic) tended to be lower (0.05 < *p* ≤ 0.10) in group C compared to group A. C18:2n6c (Linoleic) fatty acid was higher (*p* ≤ 0.01) in groups B and C compared to control group A. C20:0 (Arachidic) fatty acid tended to be higher (0.05 < *p* ≤ 0.10) in group C compared to control group A. C18:3n3 (a-Linolenic) fatty acid was higher (*p* ≤ 0.001) in groups B and C compared to control group A. C20:0 (Henicosanoic) fatty acid tended to be higher (0.05 < *p* ≤ 0.10) in group B compared to group C. Total saturated fatty acids were lower (*p* ≤ 0.05) in groups B and C compared to control group A. Total polyunsaturated fatty acids were higher (*p* ≤ 0.01) in groups B and C compared to control group A. Total n3 (omega-3) and total n6 (omega-6) fatty acids were higher (*p* ≤ 0.01) in groups B and C compared to control group A. The ratio of n6/n3 fatty acids was significantly lower (*p* ≤ 0.01) in groups B and C compared to control group A.

Table 9 describes the effect of dietary supplementation on the belly meat fatty acid profile. C16:1 (Palmitoleic) fatty acid was higher (*p* ≤ 0.05) in group C compared to the other two groups. C17:0 (Heptadecanoic) fatty acid was lower (*p* ≤ 0.01) in groups B and C compared to control group A. C18:0 (Stearic) fatty acid was lower (*p* ≤ 0.001) in group C compared to the other two groups and lower in group B compared to group A. C18:1n9c (Oleic) fatty acid was higher (*p* ≤ 0.001) in groups B and C compared to control group A. C18:3n3 (a-Linolenic) fatty acid was higher (*p* ≤ 0.01) in group B compared to the other two groups. C20:2 (cis-11,14-Eicossadienoic) fatty acid was higher (*p* ≤ 0.01) in group A compared to the other two groups and in group B compared to group C. C22:0 (Behenic) and total saturated fatty acids were higher (*p* ≤ 0.001) in group A compared to the other two groups and also higher in group B compared to group C. Total monounsaturated fatty acids were higher (*p* ≤ 0.001) in group C compared to the other two groups and also higher in group B compared to group A. Total n3 (omega-3) fatty acids were higher (*p* ≤ 0.01) in group B compared to the other two groups. The ratio of n6/n3 fatty acids was significantly lower (*p* ≤ 0.01) in groups B and C compared to control group A.

## 4. Discussion

One of the major problems facing the pork industry nowadays is the high cost of feeding, especially the price and availability of high-protein feeds [17]. During the last few years, a large effort has been made to identify and utilize new protein feed sources with high nutritional value. As a result, some insect species, such as *T. molitor* and *H.* illucence, have been tested on swine diets [40,41,42]. To our knowledge, in the present study, these meat quality parameters were investigated for the first time in pigs that were fed *T. molitor* meals.

Some studies have reported beneficial effects of *T. molitor* dietary supplementation on the performance of growing pigs, such as increased body weight and improved body weight gain [40,43]. However, another study found that supplementation of *T. molitor* at a rate of 10% of the final diet had negative effects on the growth performance of pigs [41]. The present study confirmed that *T. molitor* meal can replace other protein sources in feed like fish meal without any negative effects on bodyweight gain or carcass weight, which is in agreement with previous results [43,44,45]. It should be noted that in our case, live body weight and body weight gain were improved by the *T. molitor* meal supplementation in the first half of the trial (up to day 21), although the difference between the groups was not significant at the end of the trial (day 42). The potential explanations for these effects are unclear. Dietary chitin and its derived polysaccharides, such as chitosan, can impact human and animal gut microflora, although a more in-depth investigation on this subject is needed [46]. These compounds are considered to have “prebiotic” properties and can improve gut health and animal performance [47,48]. For example, Xu et al. [49] tested diets supplemented with chitosan in growing pigs and observed an improvement in growth performance, which they attributed to the increased growth hormone concentrations in the blood serum as well as the improved small intestinal morphological structure. Moreover, it has been hypothesized that insect meals and their PAPs affect gut microbiota differently depending on their overall protein content [46].

The nutritional, physiological, and immunological functions of the pigs can be influenced by their gut microbiota [50]. Weaning at the age of 3–4 weeks brings the young pigs face-to-face with many stressful factors (nutritional and environmental). These factors can reduce feed intake as well as nutrient digestibility and are often associated with the proliferation of pathogens such as Enterobacteriaceae [51,52]. Furthermore, one of the major factors that affects gut microbiota is diet composition, mainly the inclusion of antimicrobial compounds, either natural or synthetic [53]. It is well known that the exoskeletons of many insects are rich in chitin and other bioactive compounds known for their antimicrobial activity [15,54]. In the present experiment, bacterial populations showed that *T. molitor* supplementation reduced the total aerobes, which include numerous potentially pathogenic microorganisms [55]. At the family level, the microbiota was dominated by Lactobacillaceae, Enterococcaceae, and Enterobacteriaceae in all groups. Lactobacillaceae is a family that is generally considered to have beneficial effects on gut health [56]. These alterations can be linked to the chitin content of insect meals, which can act as a specific substrate for some gut microbiota families and thus alter the microbial fermentation metabolites that are produced in the lumen [57]. In addition, the tissues of insects are rich in bioactive peptides such as a-helical peptides (cecropins, copricin), cysteine-rich peptides (insect defensin), proline-rich peptides, glycine-rich peptides, and insect AMP-complexes that have health-promoting effects (antimicrobial, immunomodulatory, and antioxidant) in monogastric animal nutrition [15]. Liu [58] described that short- and medium-chain fatty acids and long-chain polyunsaturated fatty acids are involved in pig intestinal health by protecting against enteritis. Short-chain fatty acids promote intestinal development and the function of absorption while also enhancing the immune response independently of the gut microbiota [59]. Additionally, medium-chain fatty acids found in insect oils can replace other necessary sources of lauric acid [15]. Especially *T. molitor* larvae meals have been proposed as a rich source of unsaturated fatty acids [21].

Hematological (WBC, Lym, Mon, Gra, RBC, Hct, Hb, and THR) and most biochemical parameters (ALB, ALT, AST, CK, GLU, TBIL, and TRIG) were not affected by the addition of the two *T. molitor* meals (except for total cholesterol) in the present trial and were within the physiological reference intervals reported for swine [60]. This could be a clear biomarker of the adequate quality of the tested diets, which contributed to the maintenance of the animal’s health status. These results are in accordance with Ao et al. [19], who tested dietary *T. molitor* larvae in the diets of growing pigs. An increase in the count of blood platelets has been reported by Chia et al. [45] when they supplemented the feeds of growing pigs with 50% *T. molitor*, which may be attributed to the high digestibility of insect-based protein and high levels of minerals such as iron. Moreover, some recent studies have examined other insect meals (*H. illucens)* in broiler and pig diets and did not identify any detrimental effects on blood chemical parameters [17,61,62].

Today, there is an increasing demand for the elimination or reduction of food pathogens without the use of chemical additives [63]. Insect-derived feed materials could be an innovative solution for the elimination of chemical preservatives as they are rich in antimicrobial peptides [15]. Furthermore, Chen et al. [64] concluded that there is a correlation between gut microbiota and meat quality, indicating that the animal diet could affect the microbial populations, bacterial metabolites, and the quality of the produced meat. Similarly, Knecht et al. [65] reported that higher gut bacterial populations such as *Lactobacillus*, *Oscillibacter*, *Roseburia* spp., and *Clostridium* spp. are linked to higher meat quality. These bacteria are able to produce short- and medium-chain fatty acids as well as conjugated linoleic acid (CLA) from linoleic acid (LA), which may decrease the quantity of fat tissue in the meat [65,66]. In the present study, the microbiological analysis of shoulder and belly cuts showed that the meat of groups B and C had significantly lower counts of pathogenic bacteria such as *E. coli*, *Clostridium* spp., and *Staphylococcus* spp., which is in agreement with the conclusions of the previous authors about the connection between the microbiota of the gut of the growing pigs and the microorganisms of the meat cuts.

Regarding the quality of the meat, the pork industry spends a lot of effort to create meat products with superior quality characteristics, such as a greater nutritional value [67]. Feeding strategies are one of the major factors that can affect meat quality characteristics. Lipid oxidation is a very important indicator of meat quality as it can downgrade the nutritional properties of meat, generating toxic compounds such as MDA [63]. In the present trial, the dietary supplementation of a *T. molitor* meal did not affect the proximate composition of the different meat cuts. Concerning the meat antioxidant capacity, an increase in total phenols in all meat cuts and a reduction of TBARS (in the shoulder meat cut) were observed. Navarro Del Hierro et al. [68] reported the antioxidant potential of insect proteins from *T. molitor* larvae. Yu et al. [10] referred to the use of *H. illucens* meal in growing pigs as positively affecting the mRNA expression level of the acetyl-CoA carboxylase and the lipoprotein lipase. However, *T. molitor* supplementation in the diets of growing pigs did not affect the thiobarbituric acid-reactive substances of ham meat cuts [20]. In addition, meat color is an important acceptability parameter for consumers since they often reject products that vary from what they expect to be “normal” [31]. One of the factors that can affect pork meat color is the pigment content of the diet [69]. In the present study, meat color parameters (L*, A*, and B* values) did not differ between the treatments; therefore, the added insect meals did not affect the overall pigment content of the diets. The specific underlying mechanisms for the above effects are unknown, and the published research about the effect of insect meals on pig meat quality is still very limited. According to Yu et al. [10], there is evidence that dietary chitin and its derivatives, chitosan and chito-oligosacharides, can improve some pork meat parameters such as drip loss and color.

The fatty acid composition is another significant factor in pork meat quality. Nowadays, the benefits of PUFA, omega-3, and omega-6 fatty acids for human health have been extensively reviewed [70], and an increased intake of omega-3 fatty acids is recommended under common consumer practices [71]. It should be noted that PUFA cannot be synthesized by mammals [72]. According to Morel et al. [70], the pork meat fatty acid profile can be modified by dietary manipulation. *T. molitor* larvae have a fatty acid profile rich in monounsaturated fatty acids such as oleic, elaidic, linoleic, and eicosapentaenoic [73]. In addition, the inclusion levels of other ingredients in the diets can be modified by insect meal supplementation, thus modifying the overall amounts of dietary ether extracts or some individual fatty acids, such as modification of the soybean oil, maize, and other grains. In the present study, some differences were found in the fatty acid profiles of the shoulder and belly meat cuts of the pigs fed the two different insect meals. In the shoulder cut, lower concentrations of SFA were noted, while PUFA, omega-3, and omega-6 fatty acids were found in higher amounts. In the belly cut, SFA decreased and MUFA and omega-3 fatty acids increased with the addition of the two *T. molitor* meals. Similar results were reported by Altmann et al. [44], who observed lower concentrations of SFA and higher concentrations of PUFA in the back fat meat of growing pigs that were fed with *H. illucens* larvae instead of soybean meal. In addition, the results of the present study are in agreement with the findings of Yu et al. [10], who reported higher concentrations of omega-3 fatty acids in steak meat cut when they supplemented with *H. illucens* larvae meal in finishing pigs at two different concentrations.

## 5. Conclusions

The present study compared for the first time two different insect meals from *T. molitor*, reared either in a conventional substrate or in a substrate enriched with material from residues of medicinal aromatic plants that contain important bioactive compounds, when used as feed ingredients for growing pigs and aiming to substitute high-protein feeds such as fish meals or soybean meals. Based on the results of this feeding trial, the dietary substitution can be undertaken without any detrimental effects on animal performance or health parameters. Moreover, important quality parameters were improved in the produced meat cuts, such as the resistance to oxidation and the fatty acid profile of the meat. Presently, the use of insects as feed ingredients at the industrial level is very limited due to their low availability and high price. Furthermore, additional studies are required to better evaluate different types of insect meals and their different substitution levels in pig nutrition, as well as their long-term effects on the health of the animals. Another important consideration is the economic feasibility of large-scale production of insect meals and the global logistic networks necessary for their use.

## Figures and Tables

**Table 1:** Ingredients and chemical composition of the diets.

Ingredients, g/kg as Fed	Group A	Group B	Group C
Maize	336.0	205.4	205.4
Barley	347.0	347.0	347.0
Wheat middlings	30.0	30.0	30.0
Soybean meal (47% crude protein)	168.0	188.8	188.8
Soybean oil	19.0	54.8	54.8
Vitamin and mineral premix ^1^	60.0	60.0	60.0
Fishmeal (72% crude protein)	30.0	0.0	0.0
“Conventional” *T. molitor* meal	0.0	100.0	0.0
“Enriched” *T. molitor* meal	0.0	0.0	100.0
Benzoic acid	3.0	3.0	3.0
Zn oxide	3.0	3.0	3.0
Salt	2.0	2.0	2.0
Monocalcium phosphate (22% P)	2.0	6.0	6.0
**Calculated analysis, g/kg as fed**			
Dry matter	884.2	841.6	841.6
Digestible energy (DE, MJ/kg)	13.6	13.6	13.6
Crude protein	186.6	186.5	186.5
Crude fiber	34.5	34.9	34.9
Ether extract	39.4	79.0	79.0
Ash	52.8	54.1	54.1
Acid detergent fiber (ADF)	39.5	39.8	39.8
Neutral detergent fiber (NDF)	114.0	109.0	109.0
Total Lysine	12.3	12.2	12.2
Total Methionine and Cystine	7.7	7.4	7.4
Total Methionine	4.9	4.6	4.6
Total Cystine	2.8	2.8	2.8
Total Threonine	6.2	6.5	6.5
Total Tryptophan	2.0	2.1	2.1
Calcium	5.6	5.5	5.5
Total phosphorus	5.0	5.3	5.3
Sodium	3.0	2.9	2.9
Chloride	5.2	4.9	4.9
Potassium	6.7	6.4	6.4

^1^ Supplied per kg diet: 15,000 IU vitamin A, 50 mcg 25-hydroxycholecalciferol, 9.96 mg vitamin E, 10.02 mg vitamin K3, 3 mg vitamin B1, 10.02 mg vitamin B2, 6 mg pantothenic acid, 6 mg vitamin B6, 40.02 mcg vitamin B12, 100 mg vitamin C, 35 mg niacin, 300 mcg biotin, 1.5 mg folic acid, 375 mg choline chloride, 200 mg ferrous sulfate monohydrate, 90 mg copper sulfate pentahydrate, 60 mg manganese sulfate monohydrate, 100 mg zinc sulfate monohydrate, 2 mg calcium iodate, 300 mg sodium selenide, 150 mg L-selenomethionine—selenium, 1500 FYT 6-phytase, 80 U β-1,4-endoglucanase, 70 U β-1,3 (4)-endoglucanase, 270 U β-1,4-endoxylanase, 5000 mg benzoic acid, 40.8 mg butylated hydroxytoluene, and 3.5 mg propyl gallate.

**Table 2:** The effect of dietary *Tenebrio molitor* meal supplementation on the performance and carcass parameters in growing pigs.

Body Weight on Day (kg)	Group A	Group B	Group C	SEM	*p*-Value
1	8.41	8.51	8.42	0.138	0.950
21	14.77 ^a^	16.86 ^b^	16.04 ^ab^	0.337	0.034
42	24.86	24.98	25.29	0.478	0.934
**Weight gain for the period (kg)**					
1 to 21 days	6.36 ^a^	8.35 ^b^	7.63 ^ab^	0.254	0.003
21 to 42 days	10.09	8.13	9.25	0.400	0.131
1 to 42 days	16.45	16.48	16.88	0.433	0.909
**Feed intake per pig for the period (kg)**					
1 to 21 days	14.56	14.02	14.05	-	-
21 to 42 days	21.19	20.46	20.49	-	-
1 to 42 days	35.75	34.48	34.54	-	-
**Feed conversion ratio (FCR) for the period (kg feed/kg weight gain)**					
1 to 21 days	2.29	1.68	1.84	-	-
21 to 42 days	2.10	2.52	2.22	-	-
1 to 42 days	2.17	2.09	2.05	-	-
**Carcass parameters**					
Carcass weight (kg)	14.94 ^x^	15.66 ^xy^	16.80 ^y^	0.356	0.090
Carcass dressing percentage (%)	0.63	0.63	0.63	0.104	0.996

Group A, commercial diet; Group B, diet containing 10% “Conventional” *T. molitor* meal; Group C, diet containing 10% “Enriched” *T. molitor* meal; SEM, standard error of the mean. ^a,b^ Means (*n* = 6 per treatment) with no common superscript differ significantly (*p* ≤ 0.05). ^x,y^ Means (*n* = 6 per treatment) with no common superscript tend to (0.05 < *p* ≤ 0.10).

**Table 3:** The effect of dietary *Tenebrio molitor* meal supplementation on the intestinal microbiota populations in growing pigs.

Day 42 (Log_10_ CFU/g)	Group A	Group B	Group C	SEM	*p*-Value
Enterobacteriaceae	6.46	6.90	6.38	0.161	0.397
Enterococcaceae	4.06	4.06	4.09	0.093	0.992
Lactobacillaceae	8.12 ^y^	6.96 ^x^	7.09 ^xy^	0.273	0.084
Total aerobes	8.34 ^b^	8,63 ^b^	7,49 ^a^	0.123	<0.001
Total anaerobes	8.56	8.74	8.39	0.130	0.574

Group A, commercial diet; Group B, diet containing 10% “Conventional” *T. molitor* meal; Group C, diet containing 10% “Enriched” *T. molitor* meal; SEM, Standard error of the mean. ^a,b^ Means (*n* = 12 per treatment) with no common superscript differ significantly (*p* ≤ 0.05). ^x,y^ Means (*n* = 12 per treatment) with no common superscript tend to (0.05 < *p* ≤ 0.10).

**Table 4:** The effect of dietary *Tenebrio molitor* meal supplementation on some blood parameters in growing pigs.

Hematological Parameters	Group A	Group B	Group C	SEM	*p*-Value
WBC (m/mm^3^)	23.47	22.03	21.70	1.338	0.867
Lym (%)	34.33	35.48	37.37	1.020	0.474
Mon (%)	9.35	7.58	7.65	0.445	0.237
Gra (%)	56.32	56.93	54.98	1.247	0.818
RBC (m/mm^3^)	6.32	6.62	6.13	0.184	0.553
Hct (%)	35.02	36.32	34.28	1.131	0.692
Hb (g/dL)	11.87	12.27	11.82	0.381	0.778
THR (m/mm^3^)	329.50	325.50	296.00	14.697	0.647
**Biochemical parameters**					
ALB (g/dL)	2.63	2.57	2.47	0.837	0.721
ALT (U/L)	117.33	115.33	122.83	2.906	0.572
AST (U/L)	69.50	74.83	70.33	3.992	0.864
CHOL (mg/dL)	75.00 ^a^	70.00 ^a^	92.00 ^b^	2.753	<0.001
CK (U/L)	1189.50	1014.00	1050.67	130.476	0.862
GLU (mg/dL)	92.17	98.17	100.17	4.603	0.780
TBIL (mg/dL)	0.09	0.12	0.09	0.125	0.890
TRIG (mg/dL)	49.00	48.17	55.50	2.085	0.337

Group A, commercial diet; Group B, diet containing 10% “Conventional” *T. molitor* meal; Group C, diet containing 10% “Enriched” *T. molitor* meal; SEM, Standard error of the mean. WBC, White Blood Cells; Lym, Lymphocytes; Mon, Monocytes; Gra, Granulocytes; RBC, Red Blood Cells; Hct, Hematocrit; Hb, Hemoglobin; THR, Thrombomodulin; ALB, Albumine; ALT, Alanine aminotransferase; AST, Aspartate aminotransferase; CHOL, Cholesterol; CK, Creatine kinase; GLU, Glucose; TBIL, Total bilirubin; TRIG, Triglycerides. ^a,b^ Means (*n* = 6 per treatment) with no common superscript differ significantly (*p* ≤ 0.05).

**Table 5:** The effect of dietary *Tenebrio molitor* meal supplementation on the microbial populations of the meat in growing pigs.

Shoulder Meat Microbiota (Log_10_ CFU/g)	Group A	Group B	Group C	SEM	*p*-Value
Total microbes	5.93	5.11	5.23	0.227	0.297
*Escherichia coli*	4.27 ^b^	2.44 ^a^	4.11 ^b^	0.262	0.001
*Clostridium* spp.	3.24 ^b^	2.01 ^a^	3.25 ^b^	0.193	0.009
*Campylobacter jejuni*	3.44	2.33	3.09	0.221	0.100
*Staphylococcus* spp.	4.80 ^b^	4.61 ^a^	4.63 ^ab^	0.037	0.046
*Staphylococcus aureus*	2.60	2.46	2.38	0.061	0.348
**Belly meat microbiota (Log_10_ CFU/g)**					
Total microbes	6.04	6.20	6.33	0.128	0.854
*Escherichia coli*	4.31	3.37	3.88	0.205	0.180
*Clostridium* spp.	2.06 ^xy^	2.02 ^x^	2.81 ^y^	0.163	0.068
*Campylobacter jejuni*	3.40	2.98	2.74	0.204	0.434
*Staphylococcus* spp.	4.17	4.09	4.34	0.176	0.864
*Staphylococcus aureus*	2.40	2.46	2.18	0.158	0.780
**Boneless steak meat microbiota (Log_10_ CFU/g)**					
Total microbes	4.35	4.35	4.76	0.109	0.211
*Escherichia coli*	2.74	2.08	2.10	0.235	0.460
*Clostridium* spp.	1.45	1.47	1.61	0.087	0.751
*Campylobacter jejuni*	3.32	2.98	2.86	0.176	0.691
*Staphylococcus* spp.	3.09	2.32	2.79	0.224	0.401
*Staphylococcus aureus*	2.91	2.11	2.32	0.243	0.405

Group A, commercial diet; Group B, diet containing 10% “Conventional” *T. molitor* meal; Group C, diet containing 10% “Enriched” *T. molitor* meal; SEM, Standard error of the mean. ^a,b^ Means (*n* = 6 per treatment) with no common superscript differ significantly (*p* ≤ 0.05). ^x,y^ Means (*n* = 6 per treatment) with no common superscript tend to (0.05 < *p* ≤ 0.10).

**Table 6:** The effect of dietary *Tenebrio molitor* meal supplementation on the chemical composition of the meat in growing pigs.

Chemical Composition (%) Ham Meat	Group A	Group B	Group C	SEM	*p*-Value
Fat	2.64	3.20	3.17	0.155	0.268
Protein	19.56	20.06	19.66	0.128	0.246
Moisture	76.89	76.09	76.83	0.154	0.063
Collagen	1.02 ^b^	0.89 ^a^	0.89 ^ab^	0.025	0.050
Ash	0.98	0.97	0.95	0.322	0.931
**Boneless steak meat**					
Fat	3.18	2.57	2.77	0.141	0.191
Protein	19.80	20.61	20.26	0.144	0.488
Moisture	75.97	76.05	76.26	0.135	0.689
Collagen	1.17	1.08	1.10	0.035	0.541
Ash	1.05	0.98	0.90	0.031	0.112
**Shoulder meat**					
Fat	5.22	5.50	5.53	0.222	0.887
Protein	18.43	18.21	17.95	0.165	0.650
Moisture	75.56	75.55	75.50	0.189	0.990
Collagen	1.31	1.33	1.16	0.373	0.158
Ash	0.97	0.90	0.93	0.191	0.319
**Belly meat**					
Fat	9.87	8.61	9.54	0.277	0.152
Protein	16.93	17.55	17.10	0.164	0.303
Moisture	72.27	72.89	72.44	0.174	0.337
Collagen	1.66	1.67	1.52	0.669	0.632
Ash	1.00	0.90	0.87	0.306	0.186

Group A, commercial diet; Group B, diet containing 10% “Conventional” *T. molitor* meal; Group C, diet containing 10% “Enriched” *T. molitor* meal; SEM, Standard error of the mean. ^a,b^ Means (*n* = 6 per treatment) with no common superscript differ significantly (*p* ≤ 0.05).

**Table 7:** The effect of dietary *Tenebrio molitor* meal supplementation on the oxidative stability, pH, and color characteristics of the meat in growing pigs.

Total Phenols (g/L)	Group A	Group B	Group C	SEM	*p*-Value
Shoulder meat	1.96 ^a^	5.31 ^b^	3.85 ^ab^	0.501	0.010
Belly meat	1.83 ^x^	2.04 ^xy^	2.34 ^y^	0.097	0.084
Boneless steak meat	3.54 ^a^	5.25 ^b^	4.82 ^b^	0.265	0.023
**TBARS (mg MDA/kg)**					
Shoulder meat	0.06 ^b^	0.03 ^a^	0.05 ^ab^	0.005	0.041
Belly meat	0.052	0.048	0.049	0.002	0.758
Boneless steak meat	0.11	0.13	0.13	0.004	0.244
**pH**					
Shoulder meat	5.84	5.76	5.76	0.017	0.142
Belly meat	5.96	5.94	5.86	0.020	0.103
Boneless steak meat	5.95	5.87	6.08	0.316	0.11
Color L*					
Shoulder meat	63.22	61.50	60.22	0.910	0.432
Belly meat	64.40	58.64	61.10	1.283	0.190
Boneless steak meat	72.14	72.32	67.54	1.106	0.133
Color A*					
Shoulder meat	15.14	14.48	16.56	0.808	0.597
Belly meat	13.92	15.94	15.78	0.734	0.492
Boneless steak meat	8.08	7.14	7.60	0.588	0.831
Color B*					
Shoulder meat	12.32	13.24	12.18	0.234	0.130
Belly meat	10.12	11.54	11.22	0.571	0.602
Boneless steak meat	14.98	16.08	18.28	0.905	0.341

Group A, commercial diet; Group B, diet containing 10% “Conventional” *T. molitor* meal; Group C, diet containing 10% “Enriched” *T. molitor* meal; SEM, Standard error of the mean. ^a,b^ Means (*n* = 6 per treatment) with no common superscript differ significantly (*p* ≤ 0.05). ^x,y^ Means (*n* = 6 per treatment) with no common superscript tend to (0.05 < *p* ≤ 0.10). Lightness (L*), redness (A*), and yellowness (B*) values.

**Table 8:** The effect of dietary *Tenebrio molitor* meal supplementation on the fatty acid composition of the shoulder meat in growing pigs.

Shoulder Meat Fatty Acids	Group A	Group B	Group C	SEM	*p*-Value
C14:0 (Myristic)	0.30 ^b^	0.06 ^a^	0.17 ^a^	0.039	0.007
C15:0 (Pentadecanoic)	0.29 ^y^	0.05 ^x^	0.22 ^xy^	0.045	0.053
C15:1 (cis-10-Pentadecenoic)	2.01	1.64	1.40	0.153	0.300
C16:0 (Palmitic)	28.40	26.89	25.90	0.997	0.252
C16:1 (Palmitoleic)	0.09 ^a^	0.84 ^b^	1.48 ^b^	0.223	0.007
C17:0 (Heptadecanoic)	0.50	0.30	0.21	0.082	0.161
C17:1 (cis-10-Heptadecenoic)	0.53 ^xy^	0.82 ^y^	0.48 ^x^	0.068	0.065
C18:0 (Stearic)	12.43	10.49	11.37	0.432	0.195
C18:1n9t (Elaidic)	0.05 ^x^	0.06 ^xy^	0.09 ^y^	0.009	0.082
C18:1n9c (Oleic)	23.38 ^y^	21.78 ^xy^	20.16 ^x^	0.603	0.067
C18:2n6t (Linolelaidic)	0.06	0.07	0.07	0.008	0.959
C18:2n6c (Linoleic)	24.70 ^a^	29.28 ^b^	32.01 ^b^	1.162	0.004
C18:3n6 (γ-Linolenic)	0.07	0.05	0.08	0.007	0.340
C20:0 (Arachidic)	0.66 ^x^	1.05 ^xy^	1.16 ^y^	0.098	0.068
C18:3n3 (a-Linolenic)	0.23 ^a^	0.42 ^b^	0.47 ^b^	0.039	0.001
C20:1n9c (cis-11-Eicosenoic)	0.05	0.03	0.02	0.008	0.378
C21:0 (Henicosanoic)	0.40 ^xy^	0.56 ^y^	0.35 ^x^	0.041	0.062
C20:2 (cis-11,14-Eicossadienoic)	0.38	0.33	0.28	0.038	0.627
C22:0 (Behenic)	5.48	5.28	4.08	0.360	0.251
Σ SFA (Total Saturated)	48.47 ^b^	44.69 ^a^	43.46 ^a^	0.885	0.021
Σ MUFA (Total Monounsaturated)	26.10	25.17	23.63	0.568	0.214
Σ PUFA (Total Polyansaturated)	25.44 ^a^	30.15 ^b^	32.90 ^b^	1.194	0.005
n6 (omega 6) Fatty Acids	24.83 ^a^	29.39 ^b^	32.16 ^b^	1.161	0.004
n3 (omega 3) Fatty Acids	0.23 ^a^	0.42 ^b^	0.47 ^b^	0.039	0.001

Group A, commercial diet; Group B, diet containing 10% “Conventional” *T. molitor* meal; Group C, diet containing 10% “Enriched” *T. molitor* meal; SEM, Standard error of the mean. ^a,b^ Means (*n* = 6 per treatment) with no common superscript differ significantly (*p* ≤ 0.05). ^x,y^ Means (*n* = 6 per treatment) with no common superscript tend to (0.05 < *p* ≤ 0.10).

**Table 9:** The effect of dietary *Tenebrio molitor* meal supplementation on the fatty acid composition of the belly meat in growing pigs.

Belly Meat Fatty Acids	Group A	Group B	Group C	SEM	*p*-Value
C14:0 (Myristic)	0.31	0.29	0.10	0.049	0.170
C14:1 (Myristoleic)	0.14	0.09	0.21	0.037	0.129
C15:0 (Pentadecanoic)	0.29	0.23	0.26	0.037	0.811
C15:1 (cis-10-Pentadecenoic)	1.42	1.37	1.30	0.132	0.951
C16:0 (Palmitic)	29.37	28.09	28.43	0.301	0.212
C16:1 (Palmitoleic)	1.49 ^a^	1.79 ^a^	4.66 ^b^	0.526	0.027
C17:0 (Heptadecanoic)	0.33 ^b^	0.14 ^a^	0.20 ^a^	0.030	0.005
C17:1 (cis-10-Heptadecenoic)	0.49	0.59	0.34	0.077	0.482
C18:0 (Stearic)	14.83 ^c^	9.90 ^b^	8.01 ^a^	1.028	<0.001
C18:1n9t (Elaidic)	0.09	0.18	0.23	0.039	0.424
C18:1n9c (Oleic)	16.47 ^a^	25.12 ^b^	25.80 ^b^	1.571	0.001
C18:2n6t (Linolelaidic)	0.05	0.05	0.07	0.006	0.422
C18:2n6c (Linoleic)	28.48	27.73	28.36	0.233	0.431
C20:0 (Arachidic)	0.67	1.00	0.45	0.123	0.187
C18:3n3 (a-Linolenic)	0.45 ^a^	1.03 ^b^	0.42 ^a^	0.107	0.003
C21:0 (Henicosanoic)	0.05	0.03	0.02	0.008	0.059
C20:2 (cis-11,14-Eicossadienoic)	0.41 ^c^	0.24 ^b^	0.08 ^a^	0.052	0.003
C22:0 (Behenic)	4.23 ^c^	1.52 ^b^	0.37 ^a^	0.588	<0.001
C22:1n9 (Erucic)	0.09	0.36	0.47	0.088	0.207
Σ SFA (Total Saturated)	50.41 ^c^	41.47 ^b^	38.05 ^a^	1.863	<0.001
Σ MUFA (Total Monounsaturated)	20.20 ^a^	29.49 ^b^	33.03 ^c^	1.956	<0.001
Σ PUFA (Total Polyansaturated)	29.39	29.04	28.92	0.249	0.708
n6 (omega 6) Fatty Acids	28.53	27.78	28.42	0.233	0.426
n3 (omega 3) Fatty Acids	0.45 ^a^	1.03 ^b^	0.42 ^a^	0.107	0.003

Group A, commercial diet; Group B, diet containing 10% “Conventional” *T. molitor* meal; Group C, diet containing 10% “Enriched” *T. molitor* meal; SEM, Standard error of the mean. ^a–c^ Means (*n* = 6 per treatment) with no common superscript differ significantly (*p* ≤ 0.05).

## Data Availability

Not applicable.
